# Prevalence of HPV in the oral cavity of HIV seropositive Indian women undergoing HAART

**DOI:** 10.1186/1471-2334-14-S3-E34

**Published:** 2014-05-27

**Authors:** Jaya Mukherjee, Ritwik Dahake, Deepa Das, Gopal Sharma, Bhagyashri Purandare, Abhay Chowdhary

**Affiliations:** 1 Department of Oral Medicine Diagnosis and Radiology, YMT Dental College and Hospital, Navi Mumbai-410210, Maharashtra, India; 2 Department of Virology, Haffkine Institute, Mumbai-400012, Maharashtra, India

## Background

Oral manifestations (such as warts) of HIV infection are evident of disease progression, occurring in 30–80% of affected population. Recent evidence indicates that Human Papillomavirus (HPV) related diseases are increased in the oral cavity of HIV seropositive individuals irrespective of HAART. This study was undertaken to evaluate prevalence of oral HPV infection in Indian HIV seropositive women with/without oral warts.

## Methods

Detection of HPV was carried out from saliva of 60 women divided into equal groups of HIV seropositive with/without warts and age/sex matched healthy controls with/without warts by nested PCR using consensus primers MY09/11 and GP5+/6+ for L1 gene followed by sequencing and sequence analysis. The sequences were typed using the HPV L1 typing tool available on http://pave.niaid.nih.gov/#pavic.

## Results

Only one sample was detected to be positive for HPV (prevalence of 6.67%) and was from the HIV seropositive women with warts group. The HPV serotype was determined to be the low risk non-oncogenic HPV Type 6 by the L1 typing tool and multiple sequence alignment.

## Conclusion

While this was a preliminary study to determine prevalence of HPV in HIV seropostive women, our study has determined a positive correlation with multiple warts and oral sex as factors associated with presence of HPV. Our study also questions whether HAART has a beneficial impact on HPV induced cancer. We are of the opinion that this study is the first of its kind in Indian women and emphasize that extensive studies are necessary to establish the incidence of HPV in HIV seropositive population from India.

